# A Non-invasive Method for Assessment of Intravascular Fluid Status: Inferior Vena Cava Diameters and Collapsibility Index

**DOI:** 10.12669/pjms.324.10290

**Published:** 2016

**Authors:** Sinan Karacabey, Erkman Sanri, Ozlem Guneysel

**Affiliations:** 1 Sinan Karacabey, Assistant Professor, Emergency Department, Bozok University Faculty of Medicine, Yozgat, Turkey; 2 Erkman Sanri, MD. Emergency Medicine Department, Marmara University, Faculty of Medicine, Pendik Training and Research Hospital, Istanbul, Turkey; 3 Ozlem Guneysel Associate Professor, Emergency Department, Kartal Dr Lutfi Kırdar Training and Research Hosital, Istanbul, Turkey

**Keywords:** Ultrasonography, Intubation, End-tidal capnography

## Abstract

**Objective:**

To evaluate the correlation between central venous pressure (CVP) and inferior vena cava (IVC) diameters measured by ultrasonography (Ultrasound) in critically ill patients.

**Methods:**

Intubated critically ill patients were enrolled. The CVP values were measured using a U-tube manometer and were compared to the IVC diameters and collapsibility index, which were measured by bedside Ultrasound. Patients younger than 18 years old, who were not intubated, who had an abdominal pressure greater than 12 mmHg, and/or who were admitted for trauma were excluded from the study.

**Results:**

Eighty three patients with a mean age of 73.6±11.2 years were enrolled. The most common diagnosis was sepsis (21 patients, 25.30%). IVC inspiration measurements were statistically significantly correlated with CVP measurements (p0.05, r: 0.1). IVC collapsibility measurements showed a negative correlation with CVP measurements (p<0.01, r: 0.68).

**Conclusions:**

There is a strong correlation between CVP and IVC diameters and the collapsibility index. This is a new formula for evaluating CVP, based on our statistical analyses.

## INTRODUCTION

Fluid resuscitation in critically ill patients is a common and serious challenge.^1^ Overtreatment causes many different complications such as pulmonary edema, abdominal hypertension, and compartment syndrome.^2^ Measurements of central venous pressure (CVP), pulmonary arterial catheterization, esophageal Doppler ultrasonography (Ultrasound), and trans-esophageal echocardiography may be used to determine the volume status of critically ill patients.^3^

CVP refers to the pressure of the right atrium or superior vena cava and helps inform emergency departments and critical care units about what fluid and diuretic treatment to apply.^4^ However, hemodynamic monitorization with central venous ultrasound catheterization is limited in being costly and invasive.^5^ Considering complications such as infections, bleeding, and pneumothorax, it is better to assess fluid status using noninvasive methods.^6^

Ultrasound is a good method for noninvasive hemodynamic motorization by emergency physicians, and it may be more helpful than other noninvasive methods such as measuring urine output, pulse rate, and arterial blood pressure. New technological improvements have made Ultrasound mobile, easy to use and inexpensive.^7^

Trans-abdominal Ultrasound measurements of the IVC are noninvasive and thus are not associated with complications. In addition, many emergency departments have Ultrasound systems that can easily be used by emergency physicians.^8^ In particular, bedside Ultrasound of the IVC diameter is a useful and easy method for assessing a patient’s volume status. In the present study, we evaluated the correlation between CVP and IVC diameters as measured by Ultrasound in critically ill patients.

## METHODS

This prospective observational study was conducted at a tertiary care hospital emergency department. Our emergency department serves 360,000 patients annually with a 15% admission rate. Approximately 5% of the patients are evaluated in the critical care area. Hospital ethics committee approval was received for this study (B104ISM4340029/3). An informed consent was obtained from patients because they were intubated. A data collection form was used to gather standard data. Patient age, gender, final diagnosis, arterial blood pressure, pulse rate, CVP measurement at the end of expiration, IVC diameters during expiration and inspiration, and intra-abdominal pressure values were recorded. All the measurements were made on intubated patients.

### Selection of Participants

Between June 1 and October 25, 2012, patients who were treated in the critical care area were included in the study. All of the patients were mechanically ventilated using the Synchronized Intermittent- Mandatory Ventilation (SIMV) Volume Control mode and had a central venous line. Patients who met the following criteria were excluded: younger than 18 years old, not intubated, having had trauma, being pregnant, and/or having known or newly diagnosed intra-abdominal hypertension is defined as an intra-abdominal pressure greater than or equal to 12 mmHg.

### Methods of Measurement

**Fig.1:**
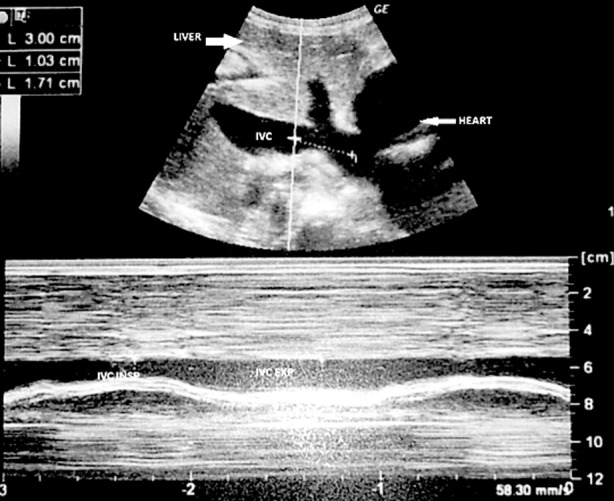
Ultrasonographic determination of inspiratory inferior vena cava (IVCinsp) and expiratory inferior vena cava (IVCexp).

The CVP, intra-abdominal pressure, and IVC diameters were measured in all patients. IVC diameters were measured by emergency department residents and specialists who received a standard basic Ultrasound course for two days. Ultrasound was performed using a Toshiba Aplio500 Ultrasound device and 3 mHz convex probe. The M-mode subxiphoidal window used to view the IVC, and both ends of the inspiration and expiration diameters were recorded in millimeters (Fig.1).

The CVP measurements were recorded from the fourth costal cartilage intersection point with the midaxillary line taken as a reference point. The CVP was measured using a U-tube manometer. The CVP results were recorded at the end of expiration in cmH_2_O.

The bladder was drained using a urinary catheter before measuring intra-abdominal pressure, and 50–100 mL normal saline was injected into the bladder; the distal portion was clamped. A 16 gauge needle was inserted into the output of the urinary catheter. The needle was connected to a three-way tap and water manometer. Symphysis pubis was taken as a reference point. After being filled with saline, the patient side of the manometer was opened. The intra-abdominal pressure results were measured and recorded in cmH_2_O and converted into mmHg (1mmHg= 1.36 cmH_2_O).^9^

### Primary Data Analysis

The NCSS Number Cruncher Statistical System 2007 and Power Analysis and Sample Size 2008 Statistical Software (NCSS, Kaysville, UT, USA) program were used for the statistical analysis of the data. Data analysis included descriptive statistics; to assess the differences between contineous variables, a Student’s t test was used. The normal distribution of variants was evaluated using Shapiro-Wilk and Kolmogorov-Smirnov analyses. Pearson and Spearman correlation analyses were used to assess correlations between CVP and IVC diameters. To assess the effects of measurements of IVC inspiration and expiration on CVP, multivariate regression analysis was used. The results were evaluated with 95% confidence intervals (CIs), and statistical significance was set at a p value less than 0.05. The data are presented as the mean ± standard deviation (SD) for continuous variables with ranges in parentheses.

## RESULTS

Eighty three patients were enrolled between June 2012 and October 2012, and 48 of these patients were male. The mean age was 73.6±11.2 years. The most common diagnosis was sepsis (n=21, 25.3%) The characteristics of patients are given in Table-I. The mean systolic blood pressure of the patients was 117.6±37.7 mmHg (range: 60–220 mmHg). The mean diastolic pressure was 70.5±24.3 mmHg (30–140 mmHg). The mean heart rate was 102.3±25.8 bpm (50–170 bpm). The mean intra-abdominal pressure was 4.8±2.3 mmHg, and there was no correlation between CVP and IAP (p>0.05). The mean IVC inspiration measurement was 14.8±5.08 mm (4.8–29 mm; median: 14.4 mm). The IVC expiration measurements varied between 10 mm and 31 mm (mean: 18.8±4.6 mm; median: 18.30 mm). The IVC collapsibility measurements varied between 0.023 and 0.567 (mean: 0.2±6.7; median: 0.1). The minimum CVP measurement was -4 cmH_2_O, and the maximum value of the CVP measurement was 30 cmH_2_O. The mean CVP measurement was 10.1±6.7. IVC inspiration measurements showed a statistically significant correlation with CVP measurements (p<0.01, r:0.53), whereas IVC expiration measurements did not (p>0.05; r: 0.1%). IVC collapsibility measurements showed a negative correlation with CVP measurements (p<0.01; r:0.68) (Table-II). In the regression analysis of the IVC measurements, the values that affected the CVP were identified using the following formula:

**Table-I:** The demographic and clinical characteristics of patients.

		Min-Max	Mean±SD
Age (years)		33-94	73.61±11.27
Systolic Pressure (mmHg)		60-220	117.60±37.77
Diastolic Pressure(mmHg)		30-140	70.57±24.38
Pulse Rate (bpm)		50-170	102.33±25.81
CVP(mmHg)		-4 - 30	10.14±6.72

		n	%

Sex	Male	48	57.8
	Female	35	42.2

*Diagnosis*		*n*	%

Acute Renal Failure		4	4.82
Acute Respiratory Failure		15	18.07
Aspiration Pneumonia		11	13.25
Gastrointestinal Bleeding		2	2.41
Intracranial Hemorrhage		6	7.23
Ischemic CerebroVascular Disease		9	10.84
PostCPR		3	3.61
Pulmonary Embolism		2	2.41
Pulmonary Edema		6	7.23
Sepsis		21	25.30
StatUltrasound EpilepticUltrasound		4	4.82

Total		83	100.00

**Table-II:** IVC measurements and CVP measurements variance and relation of CVP measurements with IVC diameters and IAP.

	Min-Max	Mean±SD	r	p
IVC inspiration	4.8 - 29	14.80±5.08	0.531	^a^ 0.001**
IVC expiration	10 - 31	18.81±4.69	0.102	^a^ 0.358
IVC Collapsibility	0.023-0.567	0.21±0.16	-0.716	^b^ 0.001***
IAP(mmHg)	0.6-9.1	4.84±2.34	-0.041	^a^ 0.716

a Pearson correlation

b Spearmann correlation

** p <0.01

CVP= 8.813+1.489(IVC Ins)+ 1.101 (IVC Exp).

The summary model of the regression model is given in Table-III. According to this model (R^2^ = 0.519), IVC inspiration and expiration measurements affected CVP at the rate of 52% (F=43.204; p<0.001).

**Table-III:** The summary and model of the IVC ins and exp measurements effect on CVP.

	Sum of Squares	Df (degrees of freedom)	Mean Square	F	p
Regression	1925.21	2	962.607	43.204	0.0001**
Difference	1782.44	80	22.281		
Total	3707.657				

R=0.721(a) R-2=0.519 Adjusted R-2=0.507 SD=4.72

## DISCUSSION

We evaluated the correlation between CVP and IVC diameters as measured by Ultrasound in intubated patients, and found a strong correlation between CVP and IVC inspiration diameter and caval index.

In general, CVP measurements, pulmonary arterial catheterization, esophageal Doppler Ultrasound, and trans-esophageal echocardiography are the most widely used methods for volume assessments of critically ill patients.^10^ However, none of these methods are perfect and all of them have both benefits and risks.^11^ In emergency departments, measurements of CVP are most commonly used. However, this procedure can cause some early and late complications. Thus a noninvasive, cost-efficient diagnostic approach to assess fluid status in emergency departments is desired.^6^

Ultrasound is a noninvasive method with broad applications in cardiac and hemodynamic evaluations. It can be used to assess acute respiratory failure, acute circulatory failure, and cardiac arrest. It can also be used for venous cannulation. Furthermore, IVC diameters can be measured by Ultrasound, and the European Association of Echocardiography recommends quantification as follows: an IVC diameter <2.1 cm that collapses >50% with a sniff suggests a normal RA pressure of 3 mmHg (0–5 mmHg); an IVC diameter >2.1 cm that collapses <50% with a sniff suggests a high RA pressure of 15 mmHg (10–20 mmHg); in scenarios in which the IVC diameter and collapse do not fit this paradigm, an intermediate value of 8 mmHg (5–10 mmHg) may be used.^12^-^15^ On the other hand, technological improvements related to Ultrasound have allowed being sought new methods. Many studies have shown that bedside Ultrasound should be performed after appropriate training and should be more solution oriented.^16^,^17^ Initially, nephrologists and cardiologists evaluated the IVC collapsibility index to assess volume status.^18^,^19^ However, Ultrasound also has some limitations. It is results are user dependent, and the image quality is affected by anatomical obstacles such as large fat deposits and bowel gas. Thus Ultrasound can be subjective.^7^

IVC is the largest vein, with a low pressure in the venous system. Dilation occurs due to changes in veous pressure. Weil^19^ first demonstrated IVC dilation in patients with right ventricular heart failure. Visualization of the IVC is easier using Ultrasound, and the values can be measured in M-mode and B-mode, an advantage over the Doppler mode^17^

Previous studies have reported that IVC expiration diameter is correlated with volume loss.^20^,^21^ However, in our study, IVC expiration diameter did not correlate with CVP. In Kusba.^20^ the volume status of patients was based on the amount of fluid sampled and blood re-infusion. In Tetsuka,^21^ it was evaluated in association with weight loss. Moreover, neither study assessed intubated patients. In another study, IVC diameters of 30 trauma patients with hemorrhagic shock were compared at admission and after fluid resuscitation. IVC diameters were a good indicator of volume loss.^22^ A subsequent study on 31 blood donors reported the same results.^19^ Lorsomradee *et al*.^23^ found a correlation between CVP and IVC diameter during cardiac surgery in patients with a CVP equal to or less than 11 mmHg. Other studies have reported correlations between CVP and IVC collapsibility.^24^,^25^ However, none of these studies assessed intubated patients.

### Limitations

There were some limitations to our study. The selection of participants was not randomized, which may have affected the results. However, subjects were enrolled without regard to the severity of illness and with no time limitations. In addition, when measuring CVP, we used a U-tube manometer instead of a piezoelectricity transducer. However, it is not easy to obtain a piezoelectricity transducer. It is an outdated method but it has a wide range of use. Finally, another limitation was that many different residents and specialists performed the procedures. Nonetheless, they were instructed about the ultrasound procedure before starting the study, and all measurements were checked by a physician.

## CONCLUSION

In intubated patients, there is strong correlation between CVP and IVC diameter during inspiration. We also found a correlation between CVP and IVC diameters and collapsibility. IVC diameter measurement can be used to assess CVP; thus, this could be an effective method for estimating the volume status of patients.
